# Authors' Reply

**DOI:** 10.1371/journal.pmed.0030166

**Published:** 2006-03-28

**Authors:** Antonio Salas, Yong-Gang Yao, Hans-Jürgen Bandelt

**Affiliations:** **1**Instituto de Medicina LegalUniversidad de Santiago de CompostelaGaliciaSpain; **2**Kunming Institute of ZoologyKunming, YunnanChina; **3**University of HamburgHamburgGermany

We gratefully acknowledge the letter by Bora Baysal [ 1], which emphasizes that there is some interesting evidence for the role of mitochondria in tumorigenesis mediated by nuclear DNA factors—an issue that was outside the scope of our article [ 2]. We, however, do not entirely agree with him that the title of our contribution [ 2] is “simply incorrect”; it could probably be described as somewhat imprecise or ambiguous. In fact, the originally submitted, more precise, title of our contribution was “A pitcher of cold water on mutational hotspots in mitochondrial DNA and the hot debate about the role of mitochondria in tumorigenesis.” In any case, the *Oxford English Dictionary*, for example, states that “reassess” is “to assess again, especially differently (derivatives: reassessment [noun])”; synonyms of assess would be “evaluate or estimate.” Certainly, the role of the mitochondria has to be reassessed since the role of their most essential element, the mitochondrial genome, remains obscure in view of dozens of studies on the potential association of tumorigenesis with mitochondrial DNA (mtDNA) that are based on obviously flawed data. Since those inadvertent circumstances (contamination and sample mix-up) are not mitochondria-specific but lab-specific, there would also be good reason to reassess other spectacular DNA findings in regard to potential laboratory errors.

We would like to stress that mtDNA somatic mutations are by no means uncommon either in normal tissues or in tumors, but the natural pattern of these somatic mutations (most commonly involving the polycytosine stretches and other well-known hotspot mutations) is quite different from those that were published in the papers criticized in our article [ 2]. Consistent with the title of our article [ 2] would be the possibility that the nuclear-mediated effect on the mitochondrial function could perhaps be mtDNA haplogroup–specific—but certainly not in the form of the artefactual instabilities, as claimed in those dubious publications (which, however, in one case, have now been explicitly defended [ 3], but unfortunately, without carrying out the necessary “forensic-type” analysis looking into potential sample mixture of the previously analyzed samples [ 4] and without determining whether the patient received blood transfusion before the onset of the disease [ 5]). Rather, some complex susceptibility background for tumorigenesis might be anticipated—in analogy to some mtDNA diseases such as Leber's hereditary optic neuropathy (LHON) [ 6].
